# Heterogeneity of human adipose blood flow

**DOI:** 10.1186/1472-6904-7-1

**Published:** 2007-01-20

**Authors:** David G Levitt

**Affiliations:** 1 Department of Integrative Biology and Physiology, University of Minnesota, 6-125 Jackson Hall, 321 Church St. S. E., Minneapolis, MN 55455, USA

## Abstract

**Background:**

The long time pharmacokinetics of highly lipid soluble compounds is dominated by blood-adipose tissue exchange and depends on the magnitude and heterogeneity of adipose blood flow. Because the adipose tissue is an infinite sink at short times (hours), the kinetics must be followed for days in order to determine if the adipose perfusion is heterogeneous. The purpose of this paper is to quantitate human adipose blood flow heterogeneity and determine its importance for human pharmacokinetics.

**Methods:**

The heterogeneity was determined using a physiologically based pharmacokinetic model (PBPK) to describe the 6 day volatile anesthetic data previously published by Yasuda et. al. The analysis uses the freely available software PKQuest and incorporates perfusion-ventilation mismatch and time dependent parameters that varied from the anesthetized to the ambulatory period. This heterogeneous adipose perfusion PBPK model was then tested by applying it to the previously published cannabidiol data of Ohlsson et. al. and the cannabinol data of Johansson et. al.

**Results:**

The volatile anesthetic kinetics at early times have only a weak dependence on adipose blood flow while at long times the pharmacokinetics are dominated by the adipose flow and are independent of muscle blood flow. At least 2 adipose compartments with different perfusion rates (0.074 and 0.014 l/kg/min) were needed to describe the anesthetic data. This heterogeneous adipose PBPK model also provided a good fit to the cannabinol data.

**Conclusion:**

Human adipose blood flow is markedly heterogeneous, varying by at least 5 fold. This heterogeneity significantly influences the long time pharmacokinetics of the volatile anesthetics and tetrahydrocannabinol. In contrast, using this same PBPK model it can be shown that the long time pharmacokinetics of the persistent lipophilic compounds (dioxins, PCBs) do not depend on adipose blood flow. The ability of the same PBPK model to describe both the anesthetic and cannabinol kinetics provides direct qualitative evidence that their kinetics are flow limited and that there is no significant adipose tissue diffusion limitation.

## Background

The physiologically based pharmacokinetic (PBPK) approach describes the drug kinetics in terms of a realistic physiological model that accurately represents the individual tissue volumes, perfusion rates and tissue/blood partition coefficients. Because adipose tissue represents from 15 to 50% of body weight, it is one of the most important factors in these models. The standard reference human adipose blood flow value of about 28 ml/kg/min [1] is based on Xenon washout measurements [2] of the local flow to a roughly 0.1 ml tissue region. These local measurements are unlikely to be representative of the whole body adipose tissue. An alternative approach is to measure the whole body kinetics of a highly lipid soluble solute, interpreting the kinetics of the slowly equilibrating compartment in terms of the average adipose perfusion [3]. This paper describes an extension of this latter approach. A detailed human PBPK model is developed which accurately describes the different organ volumes and flows and then the kinetics of highly lipid soluble solutes are used to calibrate the adipose tissue perfusion rate. In addition to determining the average adipose perfusion rate, this analysis also quantitates the heterogeneity of adipose perfusion. The adipose PBPK model derived from this analysis is then used to investigate the human pharmacokinetics of the class of persistent lipophilic solutes (e.g. dioxins, DDT, PCBs). Modeling is especially important for this class of compounds because their human pharmacokinetics cannot be accurately measured experimentally because of their very slow clearance rates.

This paper describes the PBPK modeling of a remarkable series of measurements of the pharmacokinetics of the volatile anesthetics isoflurane, sevoflurane and desflurane by Eger and colleagues [4,5]. They measured the ventilation rate and the inspired, mixed and end tidal gas concentration for 6 days following a 30 minute uptake in normal volunteers. The volatile anesthetics have two properties that make them ideally suited for studying adipose blood flow: 1) they have negligible rates of metabolism and their rate of uptake and washout from the body is determined by alveolar ventilation, which can be directly measured experimentally; and 2) their tissue/blood partition coefficients have been experimentally measured and are determined primarily by their oil/water partition and the tissue fat fraction. These two factors reduce the number of adjustable parameters in the PBPK model, increasing ones confidence in the estimates of the adipose blood flow.

The time constant (T) for adipose tissue equilibration is described by (assuming a well mixed, flow limited tissue):

T=Adipose WeightAdipose Blood Flow×(Adipose/Blood Partition Coefficient)     (1)
 MathType@MTEF@5@5@+=feaafiart1ev1aaatCvAUfKttLearuWrP9MDH5MBPbIqV92AaeXatLxBI9gBaebbnrfifHhDYfgasaacH8akY=wiFfYdH8Gipec8Eeeu0xXdbba9frFj0=OqFfea0dXdd9vqai=hGuQ8kuc9pgc9s8qqaq=dirpe0xb9q8qiLsFr0=vr0=vr0dc8meaabaqaciaacaGaaeqabaqabeGadaaakeaacqWGubavcqGH9aqpdaWcaaqaaiabdgeabjabdsgaKjabdMgaPjabdchaWjabd+gaVjabdohaZjabdwgaLjabbccaGiabdEfaxjabdwgaLjabdMgaPjabdEgaNjabdIgaOjabdsha0bqaaiabdgeabjabdsgaKjabdMgaPjabdchaWjabd+gaVjabdohaZjabdwgaLjabbccaGiabdkeacjabdYgaSjabd+gaVjabd+gaVjabdsgaKjabbccaGiabdAeagjabdYgaSjabd+gaVjabdEha3baacqGHxdaTcqGGOaakcqWGbbqqcqWGKbazcqWGPbqAcqWGWbaCcqWGVbWBcqWGZbWCcqWGLbqzcqGGVaWlcqWGcbGqcqWGSbaBcqWGVbWBcqWGVbWBcqWGKbazcqqGGaaicqWGqbaucqWGHbqycqWGYbGCcqWG0baDcqWGPbqAcqWG0baDcqWGPbqAcqWGVbWBcqWGUbGBcqqGGaaicqWGdbWqcqWGVbWBcqWGLbqzcqWGMbGzcqWGMbGzcqWGPbqAcqWGJbWycqWGPbqAcqWGLbqzcqWGUbGBcqWG0baDcqGGPaqkcaWLjaGaaCzcamaabmaabaGaeGymaedacaGLOaGaayzkaaaaaa@8C77@

For the volatile anesthetics discussed here, T is the range of about 500 min to 3 days for the estimated range of heterogeneous perfusion rates (Table 1) and partition coefficients (Table 2). The PBPK analysis is divided into two time periods: For the first time period (0 to 180 minutes) which is much less than T, all the adipose tissue behaves like an infinite sink and the analysis provides a measure of the total (or average) adipose perfusion, independent of the flow heterogeneity. During the long time period (180 min to 6 days) the adipose tissue becomes saturated and the heterogeneity of the flow becomes important. In order to resolve the flow heterogeneity it is essential to extend the pharmacokinetic measurements out to 2 or more days. Experimental human data for these times periods are very limited (Table 1). The volatile anesthetic data of Eger and colleagues [4,5] used here probably represents the most accurate and extensive available data set.

**Table 1:** Model human adipose blood flow heterogeneity.

**Solute**	**Ref**	**Ave F l/kg/min**	**F_1 _l/kg/min**	**V_1 _fract**	**F_2 _l/kg/min**	**V_2 _fract**	**F_3 _l/kg/min**	**V_2 _fract**	**Time**	**Method**
**Volatile Anesthetics**	This ms.	0.044	0.0739	0.5	0.0141	0.5			6 days	PBPK N = 2
		0.044	0.095	0.33	0.031	0.33	0.0059	0.33	6 days	PBPK N = 3
	[38]	0.071	0.213	.26	0.022	0.74			5 days	PBPK N = 2
		0.05	0.097	.41	0.019	0.59				
	[4, 5]	0.06–0.071	.11–.15	≈ 0.5	0.021–0.024	≈ 0.5			6 days	Compartmental
**Propofol**	[14]	0.042	0.042	1.0					600 min	PBPK N = 1
**Toluene**	[55]	0.032	0.032	1.0					4 days	PBPK N = 1
**Toluene**	[53]	0.024	0.042	0.4	0.013	0.5	0.0	0.1	20 hr	PBPK N = 2
**Styrene**	[54]	0.028	0.052	0.4	0.014	0.5	0.0	0.1	20 hr	PBPK N = 2

(V_i _= fraction of total adipose volume in compartment i; F_i _= perfusion rate in compartment i). (Note: in toluene [53] and styrene [54] PBPK model it is assumed that 10% of the adipose tissue has zero blood flow).

**Table 2:** Volatile anesthetic partition coefficients at 37°C.

**Solute**	**Blood/air**	**Water/air**	**Oil/air**	**Adipose/Blood**
**Isoflurane**	1.33 [56]	.544 [57]	88.2 [57]	53.2
**Sevoflurane**	0.62 [58]	.37 [59]	47 [59]	60.8
**Desflurane**	0.52 [58]	.225 [57]	17.9 [57]	27.7

The experimental data are modeled using PKQuest [6], a general, freely distributed pharmacokinetic and PBPK software routine that has now been applied to more than 25 different solutes with a wide range of pharmacokinetic properties [6-15]. A single set of optimized PBPK human parameters (eg. tissue volume, flow) has been developed that accurately describes the pharmacokinetics of a wide range of solutes. The sensitivity of the model parameters varies from solute to solute and the use of a large range of solutes provides a more stringent limit on the parameters. For example, the human muscle blood flow was determined by modeling the pharmacokinetics of D_2_O. In a previous applications of PKQuest to highly lipid soluble solutes [8,14], a single homogeneous adipose tissue model was acceptable because the experimental data did not extend to long times. In the current analysis, which extends out to 6 days, it will be shown that at least two adipose compartments with significantly different perfusion rates are required to fit the long time data.

It is assumed in PKQuest that the adipose tissue can be modeled using a well-stirred, flow limited model. This is an important assumption because it means that one can use the adipose perfusion values determined by modeling, e.g., the volatile anesthetics to predict the pharmacokinetics of any other highly lipid soluble compound, such as the dioxins or DDT. Although the majority of PBPK models of these compounds use this flow limited adipose model, there are number of research groups that have chosen to use diffusion (or permeability) limited models [16-19]. This greatly complicates and limits the application of the model since the diffusion limitation will vary in unknown ways for different solutes. The flow limited assumption is directly tested in this paper by determining whether the PBPK model could accurately describe the long time experimental human pharmacokinetics of cannabinol [20] and cannabidiol [21] using the adipose perfusion rates determined from modeling the volatile anesthetics. This should not be possible if the diffusion limited model is correct because these two classes of solutes (volatile anesthetics and cannabinoids) should have markedly different diffusion limitations.

## Methods

### General PBPK model

**Figure 1:**
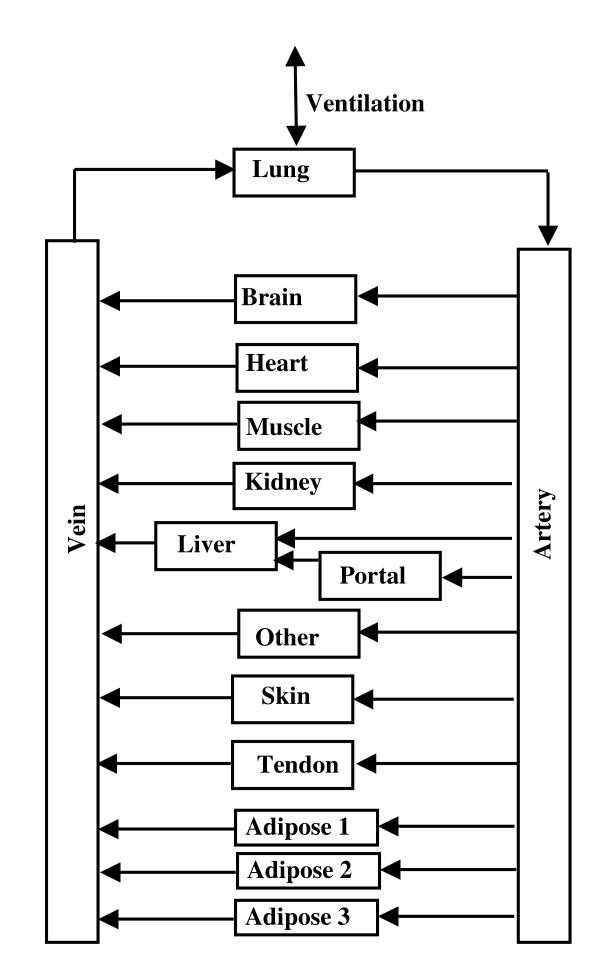
Schematic diagram of the arrangement of the different tissues in the PBPK model. The organ "portal" refers to all the organs drained by the portal vein. The connective tissue is divided between two organs: "tendon" with a relatively low blood flow and "other" with a higher blood flow. The adipose tissue was divided into N = 1, 2 or 3 equal volume compartments.

The PBPK analysis for the volatile anesthetics has been described previously [8] and the analysis for the cannabinoids is similar to that used previously for propofol [14]. The arrangement of the different tissues in the PBPK model is shown in fig. 1. The tissue parameters (blood flow, volumes, etc.) are listed in Table 3 and are similar to those used in previous applications of PKQuest [6-15]. The connective tissue is divided between two organs: "tendon" with a relatively low blood flow, and "other" with a higher blood flow. "Bone" represents inert mass with no blood flow. The actual bone blood flow is distributed among the other tissues. The major change from previous applications is that the adipose compartment has been divided into N (arbitrary) equal mass compartments. Table 3 shows the values for the 3 adipose compartment model that provided the best fit to the anesthetic data. These values are for the 70 kg, 20% fat "Standard Human". The values are scaled for different body weight and fat content. The fraction body fat in each study was determined from the subjects' average age, weight and height using the regression equation of Gallagher et. al. [22]

**Table 3:** Physiological tissue model parameters

**Tissue**	**Weight Kg**	**Perfusion l/Kg/min**	**Flow l/min**	**Fat Fraction**	**Total Fat Kg**
**Blood**	5.6			0.0075	0.042
**Liver**	1.83	0.25	0.4575	0.02	0.0366
**Portal**	1.525	0.75	1.14375	0.016	0.0244
**Muscle**	26.43	0.0225	0.594675	0.0136	0.359448
**Kidney**	0.3152	4	1.2608	0.0136	0.004287
**Brain**	1.423	0.56	0.79688	0.0176	0.025045
**Heart**	0.3355	0.8	0.2684	0.0136	0.004563
**Lung**	0.5449	0	0	0.0136	0.007411
**Skin**	2.643	0.1	0.2643	0.0136	0.035945
**Tendon**	3.05	0.01	0.0305	0.0136	0.04148
**Other**	5.616	0.02	0.11232	0.0136	0.076378
**Bone**	4.0617	0	0	0	0
**Adipose 1**	5.5419	0.095	0.5264805	0.8	4.43352
**Adipose 2**	5.5419	0.031	0.1717989	0.8	4.43352
**Adipose 3**	5.5419	0.0059	0.0326972	0.8	4.43352

**Total**	70		5.6601016		14

Several in vitro measurements of the volatile anesthetic tissue/water partition coefficients have been made [23-25]. It can be shown that the partition coefficient of tissue i can be accurately represented in terms of the fraction of fat (f_L_^i^) and water (f_W_^i^) in the tissue and the oil/water partition coefficient (K_oil-wat_) of the anesthetic [14]:

Tissue/Water=cT/cW=[fWicW+fLicL]/cW=fWi+fLiKoil−wat     (2)
 MathType@MTEF@5@5@+=feaafiart1ev1aaatCvAUfKttLearuWrP9MDH5MBPbIqV92AaeXatLxBI9gBaebbnrfifHhDYfgasaacH8akY=wiFfYdH8Gipec8Eeeu0xXdbba9frFj0=OqFfea0dXdd9vqai=hGuQ8kuc9pgc9s8qqaq=dirpe0xb9q8qiLsFr0=vr0=vr0dc8meaabaqaciaacaGaaeqabaqabeGadaaakeaacqWGubavcqWGPbqAcqWGZbWCcqWGZbWCcqWG1bqDcqWGLbqzcqGGVaWlcqWGxbWvcqWGHbqycqWG0baDcqWGLbqzcqWGYbGCcqGH9aqpcqWGJbWydaWgaaWcbaGaemivaqfabeaakiabc+caViabdogaJnaaBaaaleaacqWGxbWvaeqaaOGaeyypa0Jaei4waSLaemOzay2aa0baaSqaaiabdEfaxbqaaiabdMgaPbaakiabdogaJnaaBaaaleaacqWGxbWvaeqaaOGaey4kaSIaemOzay2aa0baaSqaaiabdYeambqaaiabdMgaPbaakiabdogaJnaaBaaaleaacqWGmbataeqaaOGaeiyxa0Laei4la8Iaem4yam2aaSbaaSqaaiabdEfaxbqabaGccqGH9aqpcqWGMbGzdaqhaaWcbaGaem4vaCfabaGaemyAaKgaaOGaey4kaSIaemOzay2aa0baaSqaaiabdYeambqaaiabdMgaPbaakiabdUealnaaBaaaleaacqWGVbWBcqWGPbqAcqWGSbaBcqGHsislcqWG3bWDcqWGHbqycqWG0baDaeqaaOGaaCzcaiaaxMaadaqadaqaaiabikdaYaGaayjkaiaawMcaaaaa@71CA@

where c_W _and c_L _are the water and lipid concentration in tissue i. It is assumed that the fat and water concentrations are in equilibrium and the tissue/blood exchange is flow limited so that the tissue and blood water concentrations (c_W_) are equal. The values of f_L _for the different tissues are listed in Table 3. They were obtained from an analysis of the experimental tissue blood partition of a series of volatile anesthetics [14]. The tissue/blood partition is then equal to:

*Tissue*/*Blood *= (*Tissue*/*Water*)/(*Blood*/*Water*)     (3)

and the blood/water partition was determined from experimental values for the volatile anesthetics (Table 2).

Since no experimental values are available for the tissue/blood partition of the cannabinoids, it was estimating from eq. (3), using eq. (2) to determine the tissue/water and blood/water partition:

Tissue/Blood=fWi+fLiKoil−watfWB+fLBKoil−wat→fLi/fLB     (4)
 MathType@MTEF@5@5@+=feaafiart1ev1aaatCvAUfKttLearuWrP9MDH5MBPbIqV92AaeXatLxBI9gBaebbnrfifHhDYfgasaacH8akY=wiFfYdH8Gipec8Eeeu0xXdbba9frFj0=OqFfea0dXdd9vqai=hGuQ8kuc9pgc9s8qqaq=dirpe0xb9q8qiLsFr0=vr0=vr0dc8meaabaqaciaacaGaaeqabaqabeGadaaakeaacqWGubavcqWGPbqAcqWGZbWCcqWGZbWCcqWG1bqDcqWGLbqzcqGGVaWlcqWGcbGqcqWGSbaBcqWGVbWBcqWGVbWBcqWGKbazcqGH9aqpdaWcaaqaaiabdAgaMnaaDaaaleaacqWGxbWvaeaacqWGPbqAaaGccqGHRaWkcqWGMbGzdaqhaaWcbaGaemitaWeabaGaemyAaKgaaOGaem4saS0aaSbaaSqaaiabd+gaVjabdMgaPjabdYgaSjabgkHiTiabdEha3jabdggaHjabdsha0bqabaaakeaacqWGMbGzdaqhaaWcbaGaem4vaCfabaGaemOqaieaaOGaey4kaSIaemOzay2aa0baaSqaaiabdYeambqaaiabdkeacbaakiabdUealnaaBaaaleaacqWGVbWBcqWGPbqAcqWGSbaBcqGHsislcqWG3bWDcqWGHbqycqWG0baDaeqaaaaakiabgkziUkabdAgaMnaaDaaaleaacqWGmbataeaacqWGPbqAaaGccqGGVaWlcqWGMbGzdaqhaaWcbaGaemitaWeabaGaemOqaieaaOGaaCzcaiaaxMaadaqadaqaaiabisda0aGaayjkaiaawMcaaaaa@7261@

The limit on the right is valid for the very large values of K_oil-wat _for the cannabinoids (24,000 or greater). In this limit the value of the tissue/blood partition depends only on the blood and tissue lipid fractions and becomes independent of the value of K_oil-wat_. The blood lipid fraction (f_L_^B^) of the cannabinoids was regarded as a model parameter that was adjusted to optimize the fit to the data.

### Volatile anesthetic data and PBPK model

The volatile anesthetic methods and data are described in 2 papers by Yasuda et. al. [4,5] that used identical methodology. The subjects average age, weight, height and calculated fat was 23 years, 72 kg and 182 cm (13.4% fat) for the sevoflurane study [5] and 25, 76 and 182 (15.4% fat) for the desflurane study [4]. Briefly, anesthesia was induced in healthy male volunteers using midazolam and/or thiopental and fentanyl and then 70% N_2_O was administered for 30 min and the ventilation was adjusted to produce normocapnea. Then, the test anesthetics (either desflurane, isoflurane and halothane [4] or sevoflurane and isoflurane [5]) were administered for 30 minutes at a nearly constant inspired concentration along with 65% N_2_O. After 30 minutes, the volatile anesthetics were discontinued, maintaining the 65% N_2_O, and the washout was measured for 150 minutes, after which the N_2_O was discontinued, the endotrachial tube removed and gas samples were obtained through a mouthpiece and non-rebreathing valve. Values of ventilation rate, inspired, mixed and end tidal concentrations were obtained at frequent intervals up to 800 minutes, and each morning thereafter for 6–7 days. Dr. Eger generously provided access to all of the original experimental data. The most important additional information that was not included in the original publications is the experimental values of the alveolar ventilation which was determined from the ventilation rate corrected for the dead space that was calculated from the inspired, mixed and end tidal partial pressures. A complete listing of the tabulated values, averaged for all subjects, is included in the Additional file 1. The halothane data was not included in the modeling because of the necessity of adding additional adjustable parameters to describe the concentration dependent halothane metabolism.

**Figure 2:**
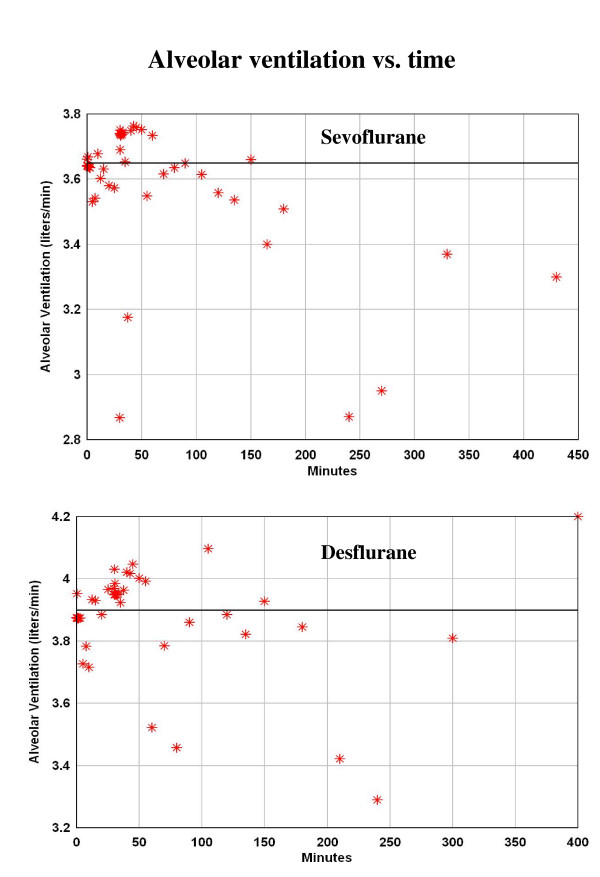
Alveolar ventilation rate for the sevoflurane (top) [5] and desflurane (bottom) [4] study. The line represents the average value for the first 180 minutes when the subjects were anesthetized.

It is necessary to introduce time dependent parameters into the PBPK analysis because the physiological conditions changed as the subject went from anesthetized to ambulatory. The analysis was divided into 3 time periods: The first period is from 0 to 180 minutes when the subjects were anesthetized and the ventilation was adjusted to produce normocapnia. The alveolar ventilation was nearly constant during this period (fig. 2) and it was assumed that it could be modeled using a constant average value. The second period was from 180 to 430 minutes when the subjects are recovering and are no longer intubated. During this period, the subjects are ambulatory, eating, etc. and the period when the ventilation is measured may not be representative of the average condition during each time interval. It is assumed that the alveolar ventilation during this period is identical to that during the first period. The last period is from 430 minutes to 6 days. During this period, the ventilation was measured once per day each morning and clearly is not representative of the average daily ventilation rate for these active young subjects. The average energy expenditure of young subjects (and the corresponding ventilation) can be as much as 1.8 times greater than the basal rate [26]. For this reason the average ventilation rate in the third period was treated as a constant parameter that was adjusted to fit the data during this time period (see Results).

It is well established that during anesthesia there are large differences between the end tidal and arterial volatile anesthetic concentration due to increased perfusion-ventilation mismatch and shunts [27-32]. As described previously [10], perfusion-ventilation mismatch is modeled in PKQuest by dividing the lung into, e.g., 16 equal volume compartments with flow and ventilation characterized by log normal standard deviations (*σ*_V _and *σ*_F_). In PKQuest, the values of *σ*_V _and *σ*_F _are described by parameters that have a value = 1 for normal ambulatory humans. During the first period, *σ *(= *σ*_V _= *σ*_F_) is adjusted to fit the observed end alveolar concentration (see Results). It is assumed that *σ *is reduced by a factor of 2 during the second period and back to the normal value during the last period. In addition, a venous-arterial shunt was included which was set to 8% during the first (anesthetized) period [29], and then reduced to the normal value of 2% during the second two periods. It is assumed that there was no significant metabolism or skin loss of the anesthetics, consistent with the 100% 6 day ventilatory recovery analysis of Yasuda et. al. [4,5]. In order to scale for differences in inspired concentration (constant during 30 minute uptake), all plots are in terms of P/P_insp _where P is the end tidal partial pressure.

In order to characterize the adipose flow heterogeneity the adipose tissue was divided into N (= 1, 2 or 3) equal volumes with flows adjusted to optimally fit the data and the quality of the model fit was determined for each value of N. The adipose tissue compartments (exchange time constants > 500 minutes) will be far from saturation during the first time period (0 to 180 minutes). During this period the adipose tissue behaves like an infinite sink so that the kinetics depend only on the total adipose blood flow and are relatively independent of the heterogeneity. The total adipose blood flow was determined by optimizing the fit to this early time data using a homogeneous (N = 1) adipose model. For the N adipose compartments, there were N adjustable parameters representing: 1) the total adipose blood flow (determined from fitting the N = 1 model to the short time data) and 2) the fraction of the total flow in the first N-1 compartments (determined from fitting the long time data). In addition, there where two other adjustable parameters: 1) the value of perfusion ventilation mismatch (*σ*) during the first period, and 2) the average ventilation rate in the last period. All other parameters were determined either from experimental measurements (Table 2) or use of the previously derived "Standard Human" PBPK model (Table 3). The identical parameter set was used for all 3 anesthetics (and 4 data sets since there were two sets of isoflurane data). The optimal parameter set was determined by finding the set of values that gave the best fit as measured by the average weighted residual error (WRE = abs((model-exp))/model). In addition, the weighted residual sum of squares (WRSS = sum [sqr{(model-exp)/model}] was determined. The Akaike criterion (= Nln(WRSS)+2p, N = # of data points, p = # of parameters) was used to compare the different values of N [33]. The values of the blood/water, blood/air and oil/water partition coefficients were determined from the experimental value of blood/air (Kbair), water/air (Kwair) and oil/air (Koair) listed in Table 2. The tissue/blood partition (Table 2) was determined from eqs. (2) and (3) with the tissue lipid fraction (f_L_^i^) listed in Table 3.

### Cannabinoid data and PBPK model

The data and experimental methodology is described in the cannabinol [20] and cannabidiol [21] publications. Deuterium labeled compounds were administered to normal young male volunteers either by smoking or by a 2 min 20 mg IV infusion and plasma values were followed for 3 days by GC/MS. The subjects average age, weight, height and calculated percent fat was 25 years, 72.17 kg and 178.8 cm (15.1% fat) for the cannabinol study and 26.4, 78.6 and 183 (16.1% fat) for the cannabidiol study. A constant set of PBPK parameters was used for the 3 days. Only the IV data was used.

The heterogeneous adipose model obtained from modeling the volatile anesthetic data was used unchanged for the cannabinoids. The values of the oil/water partition coefficients listed in Table 4 were determined by extrapolation from the octanol/water values (see Additional file 2 for details). Only two adjustable parameters were used to optimize the model fit to this data: 1) the liver cannabinoid clearance; and 2) the fat fraction of blood (f_L_^B^) which was used (eq. (4)) to determined the tissue/blood partition coefficient. The optimal value of f_L_^B ^was 0.0075 and the corresponding values of the blood/water (eq. (2)) and adipose/blood (eq. (4)) partition coefficients are listed in Table 4.

**Table 4:** Cannabinoid partition coefficients

**Solute**	**Oil/Water**	**Blood/water**	**Adipose/Blood**
**Cannabinol**	257,000	1928	106.6
**Cannibidiol**	24,000	181	106.1

## Results

### Early time volatile anesthetic homogeneous (N = 1) adipose PBPK model

This section describes the model fitting during the period the subjects were anesthetized (0 to 180 minutes). During this period, the alveolar ventilation was nearly constant (fig. 2) and a constant value was used for this period equal to the average experimental value. The average value was 3.65 liters/min for the sevoflurane study (fig. 2, top) and 3.9 liters/min for the desflurane study (fig. 2, bottom). This difference is roughly proportional to the difference in the average body weights for the two studies (72 versus 76 kg).

**Figure 3:**
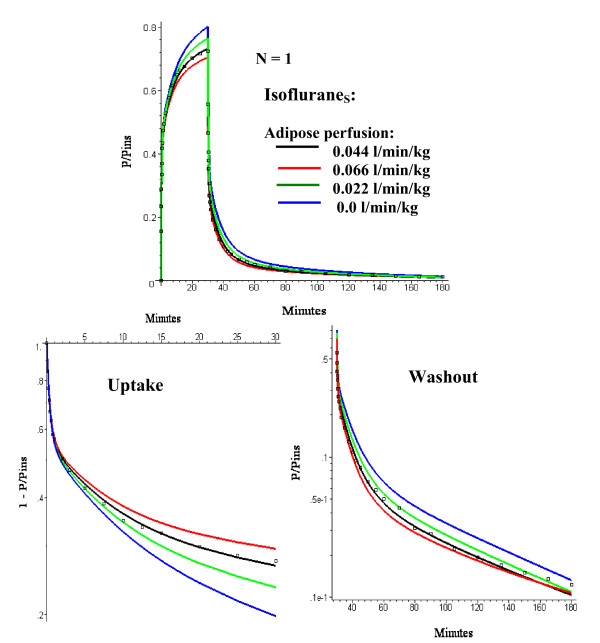
Comparison of Isoflurane_S _early time experimental data (squares) and model predictions (line) for the one adipose compartment (N = 1) PBPK model for adipose perfusion rates (F_T_) varying from 0 to 0.066 l/kg/min. The optimal value of *σ *(= 3) is used. The top panel shows an absolute plot of the (end tidal partial pressure)/(inspired partial pressure) (= P/P_insp_) versus time. The bottom panels show semi log plots of 1 - P/P_insp _during the 30 minute uptake period (left) and P/Pinsp (right) during the 150 minute washout period.

**Figure 4:**
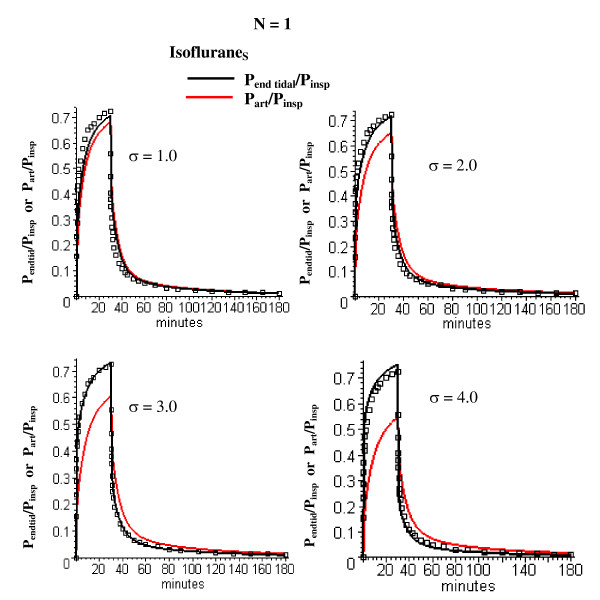
Comparison of Isoflurane_S _early time experimental data (squares) and model predictions for the one adipose compartment (N = 1) PBPK model for *σ *(measure of perfusion-ventilation mismatch) varying from 1 (normal value) to 4. The optimal value of F_T _(= 0.044 l/kg/min) is used. The end tidal/inspired (P_end tid_/P_insp_, black) and arterial/inspired (P_art_/P_insp_, red) partial pressures are shown.

**Figure 5:**
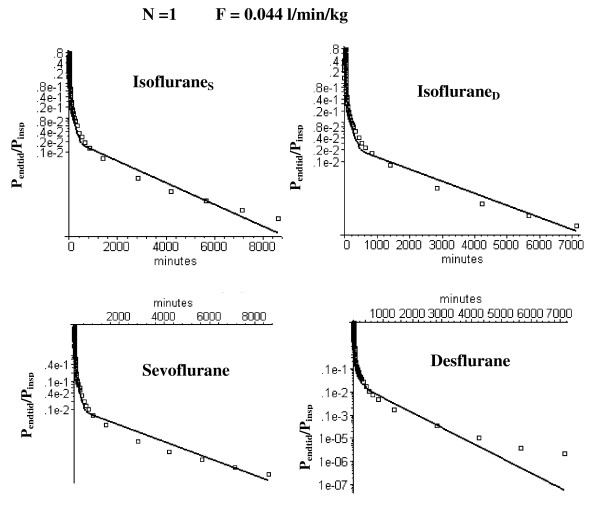
Semi log plots of comparison of the long time washout data (squares) versus the N = 1 PBPK model (solid line) using the optimal value of F_T _(= 0.044 l/kg/min) determined from the short time modeling analysis (fig. 3). Results are shown for data from the two studies by Yasuda et. al. in which sevoflurane and isoflurane (= Isoflurane_S_) [5] were compared and desflurane and isoflurane (= Isoflurane_D_) [4] were compared.

**Figure 6:**
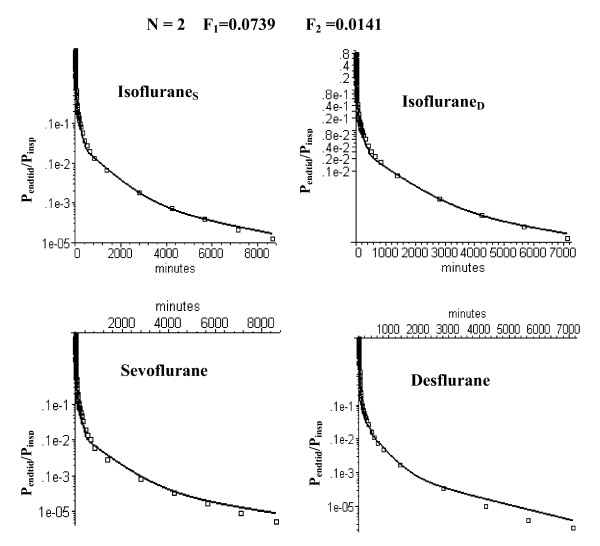
Semi log plots of comparison of the long time washout data (squares) versus the PBPK model with two equal volume adipose compartments with optimal perfusion rates of 0.0739 and 0.0141 l/kg/min, respectively.

**Figure 7:**
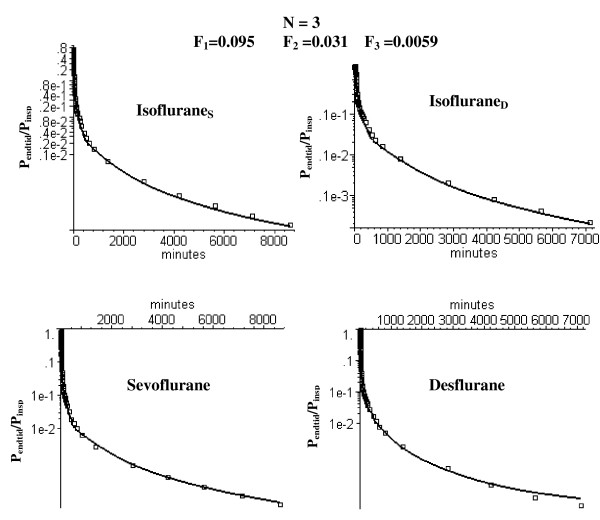
Semi log plots of comparison of the long time washout data (squares) versus the PBPK model with three equal volume adipose compartments with optimal perfusion rates of 0.095, 0.031 and 0.0059 l/kg/min, respectively.

**Figure 8:**
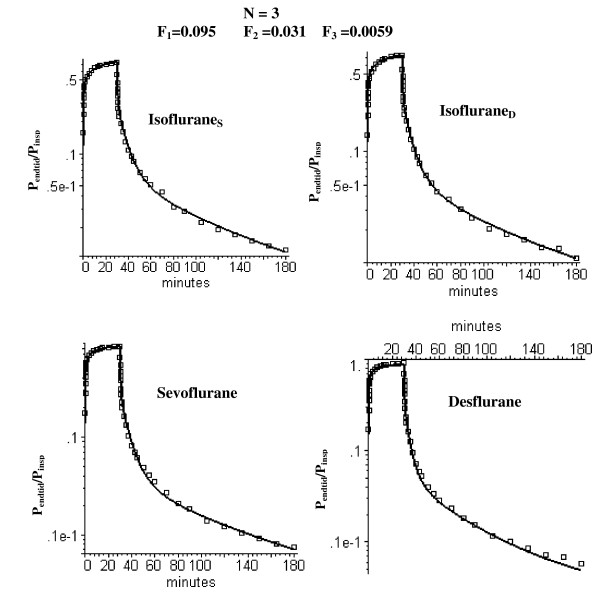
Semi log plots of comparison of the short time uptake and washout data (squares) versus the PBPK model with three equal volume adipose compartments (N = 3) with optimal perfusion rates of 0.095, 0.031 and 0.0059 l/kg/min, respectively.

As discussed above, the kinetics for this early period depend primarily on the total adipose blood flow and are relatively independent of the adipose heterogeneity. Thus for this period one can use a single adipose compartment and there are only two adjustable parameters: 1) the average adipose tissue perfusion rate (F_T_); and 2) the value of *σ *which characterizes the perfusion ventilation mismatch during this period when the subjects are anesthetized. The optimal values were obtained by minimizing the WRE for the four data sets (Sevoflurane, Desflurane, Isoflurane_S _and Isoflurane_D_) where the subscripts S and D refer to the sevoflurane and desflurane study, respectively. It was found by trial and error that parameters that optimized the fit to Isoflurane_S _were usually optimal for the entire data set. In this section, only the fits to the Isoflurane_S _data (figs. 3 and 4) are shown. See figs. 5, 6, 7, 8 for the model fits for the other solutes.

The optimal fit was for an F_T _of 0.044 liters/kg/min and a *σ *= 3 (where *σ *= 1 is the value for normal humans [10]). Figure 3 shows the model fits to the Isoflurane_S _data with *σ *= 3 and F_T _varying from 0 to 1.5 times the optimal value. In order to emphasize the quality of the fit, the data has been plotted both on an absolute scale (top) and as the semi log plots of 1 - P/P_insp _during uptake (bottom, left) and P/P_insp _during washout (bottom, right). (P and P_insp _refer to the end tidal and inspired partial pressure, respectively).

Figure 4 shows the experimental Isoflurane_S _end tidal data and the model end tidal and arterial concentrations using the optimal F_T _(= 0.044) and *σ *= *σ*_V _= *σ*_F _varying from the normal human value (= 1) to 4 times the normal value (= 4). For the optimal value (*σ *= 3) during this anesthetized period, the end tidal partial pressure is about 17% greater than the arterial during uptake.

### Long time volatile anesthetic pharmacokinetics and the N compartment adipose PBPK model

Figure 5 shows that the optimal homogeneous (N = 1) model discussed above (F_T _= 0.044) provides a poor fit to the long time (6 day) anesthetic experimental data. As discussed in the Methods, heterogeneity of the adipose blood flow was characterized by dividing it into N equal weight compartments and determining the flow in each compartment that optimized the fit to the experimental data. As the number of adipose tissue compartments is increased, it is essential to keep the total flow (i.e. average perfusion rate F_T _= 0.044 l/min/kg) fixed in order to maintain the fit to the early time data. Thus, there is one additional adjustable parameter for the N = 2 case (the fraction of the total flow in the first compartment) and two additional parameters for the N = 3 case (the fraction of the total flow in compartments 1 and 2). Figure 6 shows the semi-log plots of the optimal fit to the data for the N = 2 case. The semi-log plots for the N = 3 case are shown in fig. 7 (long time) and fig. 8 (short time). Table 1 (top 2 rows) summarizes the perfusion rates in the different adipose compartments for the N = 2 and N = 3 models and Table 5 lists the value of the WRE, WRSS and the Akaike number for the different solutes, time periods and value of N.

**Table 5:** Weighted residual error (WRE), weighted residual sum of squares (WRSS) and Akaike number for each solute, adipose model and time period

**Solute**	**Time period**	**Data (# pts)**	**N = 1**			**N = 2**			**N = 3**		
			**WRE**	**WRSS**	**Akaike**	**WRE**	**WRSS**	**Akaike**	**WRE**	**WRSS**	**Akaike**
**Isoflurane_S**	1	45	0.062	0.329	-50.0264	0.0572	0.285	-54.487	0.0575	0.284	-52.6451
	2	5	0.41	1.004	0.01996	0.0224	0.329	-3.55849	0.169	0.182	-4.51874
	3	10	0.44	3.94	13.71181	0.12	0.24	-12.2712	0.125	0.226	-10.8722
**Isoflurane_D**	1	45	0.047	0.186	-75.6904	0.0385	0.116	-94.9374	0.0375	0.11	-95.3274
	2	6	0.37	1.044	0.258357	0.189	0.285	-5.5316	0.131	0.14	-7.79668
	3	9	0.32	1.096	0.825005	0.127	0.248	-10.5489	0.119	0.16	-12.4932
**Sevoflurane**	1	45	0.0729	0.386	-42.8363	0.0664	0.332	-47.6179	0.0648	0.32	-47.2745
	2	5	0.39	0.955	-0.23022	0.242	0.364	-3.05301	0.192	0.225	-3.45827
	3	10	0.36	1.657	5.050087	0.215	0.573	-3.5687	0.103	0.171	-13.6609
**Desflurane**	1	45	0.0869	0.866	-6.47417	0.0586	0.32	-49.2745	0.0477	0.195	-69.564
	2	6	0.323	0.768	-1.58379	0.167	0.204	-7.53781	0.119	0.114	-9.02934
	3	9	5.08	1416	65.30032	0.196	0.641	-2.00253	0.139	0.338	-5.76238

**Figure 9:**
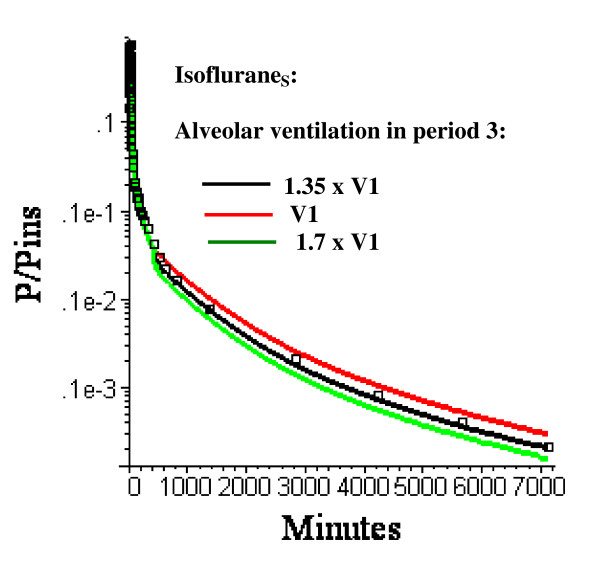
Semi log plots of the Isoflurane_S _long time washout data (squares) versus the PBPK model with three equal volume adipose compartments (N = 3) with optimal perfusion rates and alveolar ventilation during period 3 (430 minutes to 6 days) varying from V1 (= 3.65 l/min = average value during anesthetized period) to 1.7 × V1.

As described in the Methods, the parameters vary as function time. During the last time (430 minutes to 6 days) when the subject is ambulatory, *σ *is set to 1 (normal value) and the average alveolar ventilation (V_3_) is regarded as an adjustable parameter. In figs. 5, 6, 7, 8, the alveolar ventilation in the last period (V_3_) is 1.35 (the optimal value for the N = 3 model) times the experimental value for the first period (V_1_). Figure 9 shows the relatively small influence of variations in this parameter (V_3_) for Isoflurane_S_.

**Figure 10:**
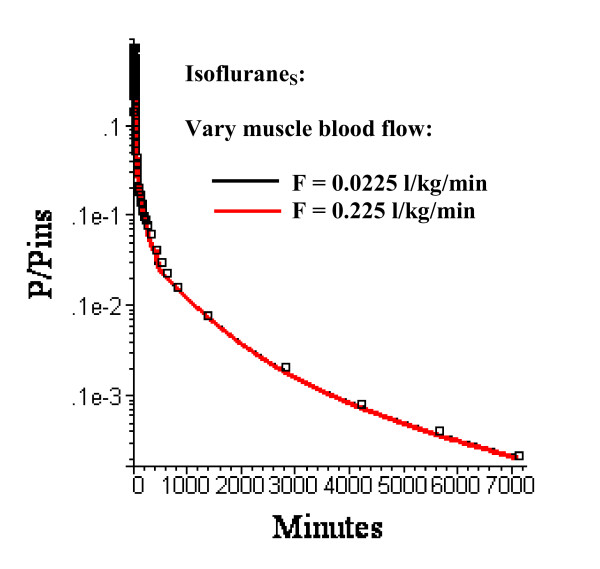
Semi log plots of the Isoflurane_S _long time washout data (squares) versus the PBPK model with three equal volume adipose compartments (N = 3) with optimal adipose perfusion rates and muscle perfusion rates in period 3 (430 minutes to 6 days) of 0.0225 l/kg/min (black line, the "Standard" resting human value) or 0.225 l/kg/min (red line). The difference in the model results for the 10 fold change in muscle blood flow is so small that the two curves are superimposed.

**Figure 11:**
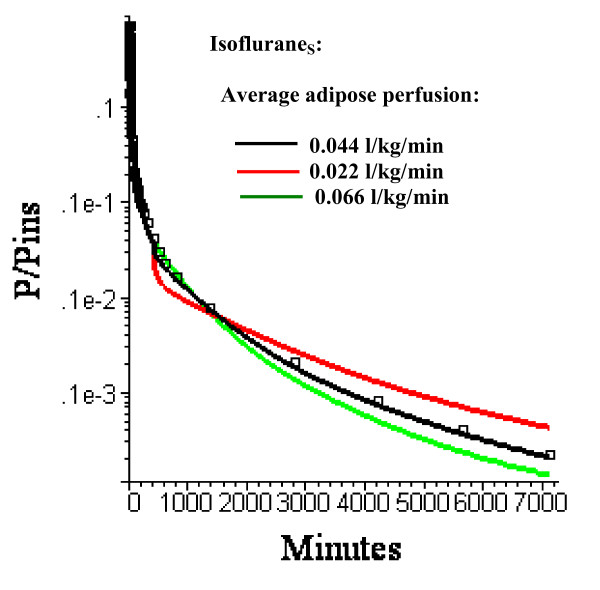
Semi log plots of the long time washout data (squares) versus the PBPK model with three equal volume adipose compartments (N = 3) with average adipose perfusion rates of 0.022 (red), 0.044 (black, the optimal value) or 0.066 l/kg/min. The relative perfusion rates among the 3 compartments are fixed at the optimal values.

It is assumed that cardiac output and blood tissue distribution during the last time period is equal to the resting "Standard Human" set of values (Table 3). Actually, one would expect that during this ambulatory period there would be significant increases in, e.g., muscle flow. However, as shown in fig. 10, even a 10 fold increase in muscle blood flow (from 0.0225 to 0.225 l/kg/min) with an accompanying doubling of cardiac output (from 6.36 to 12.61 l/min) during this period has a negligible effect on the pharmacokinetics. In contrast, changes in adipose blood flow in this period (keeping the same relative distribution to the 3 adipose regions) have dramatic effects on the kinetics (fig. 11).

### PBPK model for cannabinol and cannabidiol

**Figure 12:**
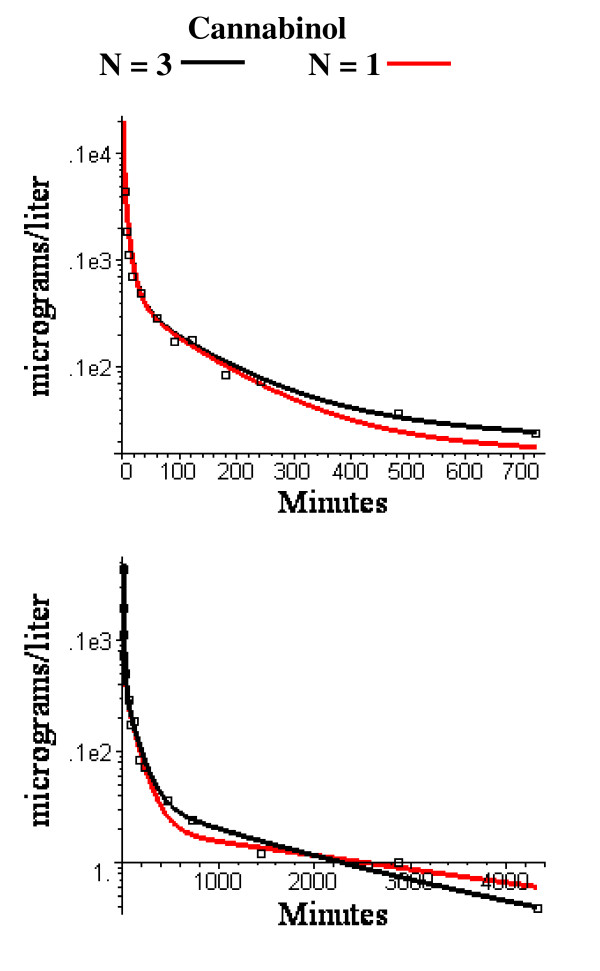
Semi log plots of short (top) and long time (bottom) experimental washout data for cannabinol versus the PBPK model using the identical N = 3 (black) or N = 1 (red) adipose compartment flow rates determined from the volatile anesthetic data.

**Figure 13:**
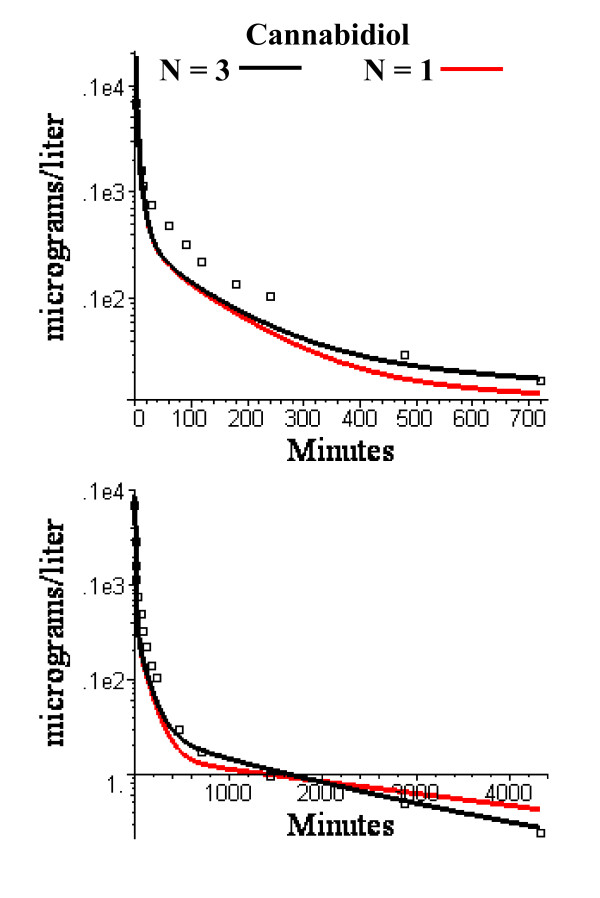
Semi log plots of short (top) and long time (bottom) experimental washout data for cannabidiol versus the PBPK model using the identical N = 3 (black) or N = 1 (red) adipose compartment flow rates determined from the volatile anesthetic data.

The N = 1 and N = 3 compartment PBPK adipose model that provided the best fit to the anesthetic data was used unchanged for the cannabinoid data. There are two adjustable parameters in the cannabinoid PBPK model: 1) the liver clearance for each cannabinoid, and 2) the value of the fraction of lipid in blood (f_L_^B^) which is used to determine the tissue/blood partition coefficient (eq. (4) and was assumed to be identical for both cannabiniods. In selecting the parameters, the long time points (480 minutes to 3 days) were heavily weighted because these time points have the strongest dependence on the adipose blood flow. The optimal value of f_L_^B ^was 0.0075. This is close to the reported value of normal human blood total lipid of 0.0082 gm/ml [34]. The optimal values of the liver clearance was 1.0 l/min for cannabinol (total liver blood flow = 1.78 l/min) and 1.3 l/min for cannabidiol (total liver flow = 1.91 l/min). Semi log plots of the short and long time model fits are shown in figs. 12 and 13. The WRE was 0.18 for cannabinol and 0.45 for cannabidiol.

## Discussion

### Heterogeneity of adipose blood flow

As discussed above, for solutes with a high fat solubility (i.e. fat/water partition of 50 or greater) the adipose tissue behaves at short times like an infinite sink so that the only important parameter is the total adipose blood flow and the pharmacokinetics do not depend on either the fat volume or the distribution of adipose blood flow. As the measurement time period extends out to the time of the adipose/blood exchange time constant, the adipose tissue becomes saturated and the flow heterogeneity becomes important. This can be seen by comparing the homogeneous (N = 1) and heterogeneous (N = 2 and N = 3) models at short (figs. 3 and 8) and long (figs. 5, 6 and 7) times. At short times (0 to 180 min, period 1), the N = 1 and N = 3 model fits (and weighted residual error, Table 5) are nearly identical while at long times (430 min to 6 days, period 3) the homogeneous (N = 1) model provides a poor fit to the data, with a weighted residual error more then 3 times larger than the N = 3 model (Table 5). Based on the Akaike criteria for time period 3 (best model is the one with lowest Akaike number), the N = 2 compartment model is superior for Isoflurane_S _while the N = 3 model is better for the other 3 data sets (Table 5). The differences between the N = 2 (fig. 6) and N = 3 (figs. 7 and 8) compartment model are small and are probably not significant.

The confidence one has in this PBPK model estimate of adipose perfusion heterogeneity is, to a large extent, subjective. Certainly, the smaller the number of adjustable parameters, the more likely the model is correct. There are 4 adjustable parameters in the N = 2 model: 1) *σ *– the value of the perfusion-ventilation mismatch during the early anesthetized time period (0 to 180 minutes); 2) F_T _– the total average adipose blood flow (determined by fitting the N = 1 model to the early time data); 3) F_1 _– the fraction of blood flow in the first adipose compartment; and 4) V_3 _– the alveolar ventilation during the 1 to 6 day ambulatory period relative to that during the anesthetized period. The N = 3 model has one additional parameter (the fraction of flow in the second compartment). All the other PBPK parameters use experimental measurements (i.e. blood/air partition) or the previously derived "Standard Human" PBPK parameter set. The value of *σ *determines the relationship between the end tidal and arterial partial pressure. The model value of *σ *= 3 corresponds to an end tidal partial pressure about 17% greater than the arterial pressure after 30 minutes uptake (fig. 4). This is nearly identical to the experimental result of Eger and Bahlman [32] in a similar set of subjects (normal young volunteers). The model has only a weak dependence on V_3 _(fig. 9) and the model value of 1.3 times the resting value is reasonable. It should be emphasized that the identical set of these 4 adjustable parameters were used to fit 4 different sets of experimental data (influrane_S_, influrane_D_, sevoflurane and desflurane). The good fit to 3 solutes with significantly different partition values (Table 2) lends further support to the validity of the model.

At short times (0 to 180 min), the non-adipose tissues dominate the kinetics, as seen by comparing the 0.044 l/min/kg flow versus the zero adipose blood flow (fig. 3). In contrast, at long times, about 95% of the volatile anesthetic is contained in fat and the adipose tissue dominates the pharmacokinetics. For example, in the third time period (430 minutes to 6 days) a 10 fold increase in muscle blood flow (and a corresponding 2 fold increase in cardiac output) has no significant effect on the pharmacokinetics (fig. 10).

Table 1 compares the current results with previous estimates of adipose blood flow using lipid soluble solutes. Using the same data as used in this study, Yasuda et. al [4,5] estimated the adipose perfusion heterogeneity by applying a compartmental or "mammillary" model approach. They assumed that the kinetics could be described by a 5 compartment model whose rate constants are determined by exponential curve fitting. Making assumptions about each compartment's tissue/blood partition, these rate constants can be interpreted in terms of volumes and flows. This approach is more general then the PBPK model because it does not make any a priori assumptions about tissue volumes or perfusion. However, this is also a major weakness because, for example, the total adipose volume and the total tissue volume do not necessarily equal the known body fat or total body volume and the "central compartment" volume may differ significantly from the known blood volume. The reproducibility of these parameters depends on the 5 compartments having clearly separable time constants. Yasuda et. al. assumed that two of the 5 compartments were adipose. The volumes of the two adipose compartments were roughly similar and the perfusion rates of the slow and fast adipose compartment were in the range of 0.022 and 0.12 l/kg/min (Table 1).

The PBPK approach used is here is closely related to the modeling of the volatile anesthetic experimental data of Carpenter et. al. [35-37] by Fiserova-Bergerova [38]. However, there are several important differences: 1) The experimental subjects in the Carpenter et. al. studies were undergoing nephrectomy with the possibility of confounding changes in PBPK parameters. In contrast, there was no surgical intervention for the subjects used in current analysis [4,5]. 2) The experimental values of the alveolar ventilation values were not available to Fiserova-Bergerova and his assumed value of 5 l/min is about 35% larger than the experimental value in the current studies. 3) There was no correction for perfusion/ventilation mismatch or changes in PBPK parameters between the anesthetized and ambulatory periods.

The average value of the PBPK adipose flow estimate in the current study (0.044 l/kg/min, Table 1) is in good agreement with more direct human measurements which, of necessity, represent specific local values. The ^133^Xe washout method yields values for the abdominal adipose flow ranging from 0.025 to 0.035 l/min/kg [39-43]. Using the [^15^O]-labeled water method, Virtanen obtained higher values of 0.045 (perienal fat) to 0.059 l/min/kg (visceral fat) [44].

All the model analyses of the lipid soluble compounds have concluded that at least two compartments with large volumes of distributions and significantly different time constants are necessary to describe the long time kinetics (Table 1). These two compartments almost certainly contain large fractions of fat. For example, if these compartments represented muscle, they would have to have volumes 30 times the assigned adipose volumes and perfusion rates 1/30 of adipose because the tissue/blood partition of adipose tissue is about 30 times that of muscle [24]. However, the specific physiological interpretation of the two compartments is more ambiguous. For the N = 2 model, the two adipose perfusion rates differ by a factor of 5.2 (Table 1). Measurements of adipose blood flow in rats using microspheres find about a 5 fold perfusion range for different fat depots [45]. However direct methods in humans do not find large perfusion differences in different adipose tissues (subcutaneous, visceral and perirenal [44] or abdominal and femoral [40]). It cannot be determined from this PBPK analysis whether the heterogeneity represents a macro or microscopic heterogeneity. Eger et. al. [46] suggested that the high flow adipose compartment might be an artifact of intertissue diffusion from high blood flow tissues (e.g. kidney, intestine, dermis) to surrounding adipose tissue. This explanation does not seem to be consistent with the cannabinoid results, as discussed below in the "diffusion limited" section.

### Pharmacokinetics of persistent lipophilic compounds (Dioxins, DDT, PCBs)

The pharmacokinetics of this class of compounds is characterized by extremely slow excretion rates, with time constants of years [47-50]. The use of PBPK models for this solute class is one of the most important applications of PBPK modeling because of the difficulty of measuring the human pharmacokinetics for solutes with these long time constants. Fortunately, there are some important simplifications in the PBPK model for this solute class that greatly increase ones confidence in these models.

Firstly, these persistent compounds all have K_oct-wat _of about 10^6^or greater and the K_oil-wat _should be similar (see Additional file 2). This means that the tissue/blood partition coefficient can be described by eq. (5) (the limit of eq. (4)) and depends only on the tissue and blood lipid fraction (f_L_) and is independent of the actual value of K_oil-wat_.

Tissue/Blood=fLi/fLB     (5)
 MathType@MTEF@5@5@+=feaafiart1ev1aaatCvAUfKttLearuWrP9MDH5MBPbIqV92AaeXatLxBI9gBaebbnrfifHhDYfgasaacH8akY=wiFfYdH8Gipec8Eeeu0xXdbba9frFj0=OqFfea0dXdd9vqai=hGuQ8kuc9pgc9s8qqaq=dirpe0xb9q8qiLsFr0=vr0=vr0dc8meaabaqaciaacaGaaeqabaqabeGadaaakeaacqWGubavcqWGPbqAcqWGZbWCcqWGZbWCcqWG1bqDcqWGLbqzcqGGVaWlcqWGcbGqcqWGSbaBcqWGVbWBcqWGVbWBcqWGKbazcqGH9aqpcqWGMbGzdaqhaaWcbaGaemitaWeabaGaemyAaKgaaOGaei4la8IaemOzay2aa0baaSqaaiabdYeambqaaiabdkeacbaakiaaxMaacaWLjaWaaeWaaeaacqaI1aqnaiaawIcacaGLPaaaaaa@49C2@

This is a major advantage since the value of the tissue/blood partition coefficient is the major source of uncertainty in most PBPK modeling.

**Figure 14:**
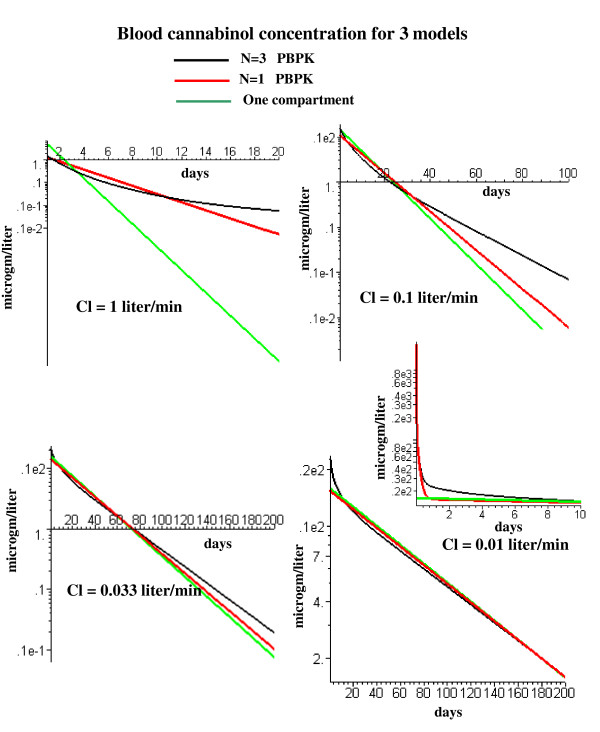
Semi log plots of long time cannabinol washout for the simple one well mixed compartment model (green) and the PBPK N = 1 (red) and N = 3 (black) adipose models. Plots are shown for liver cannabinol clearance (Cl) varying from 1.0 l/min (the optimal value for cannabinol) down to 0.01 l/min. The inset shows the early time result for the 0.01 l/min clearance.

Secondly, if the liver clearance time constant is long compared to the adipose exchange time constant, the liver clearance becomes rate limiting and the kinetics at long times are no longer dependent on the adipose (or any other tissue) blood flow. In this limit, the blood concentration (C(t)) can be described by a simple well stirred one compartment model:

C(t)=C0exp⁡(−t/T)T=Veq/ClC0=Dose/Veq     (6)
 MathType@MTEF@5@5@+=feaafiart1ev1aaatCvAUfKttLearuWrP9MDH5MBPbIqV92AaeXatLxBI9gBaebbnrfifHhDYfgasaacH8akY=wiFfYdH8Gipec8Eeeu0xXdbba9frFj0=OqFfea0dXdd9vqai=hGuQ8kuc9pgc9s8qqaq=dirpe0xb9q8qiLsFr0=vr0=vr0dc8meaabaqaciaacaGaaeqabaqabeGadaaakeaafaqaaeGabaaabaGaem4qamKaeiikaGIaemiDaqNaeiykaKIaeyypa0Jaem4qam0aaSbaaSqaaiabicdaWaqabaGccyGGLbqzcqGG4baEcqGGWbaCcqGGOaakcqGHsislcqWG0baDcqGGVaWlcqWGubavcqGGPaqkaeaafaqabeqacaaabaGaemivaqLaeyypa0JaemOvay1aaSbaaSqaaiabdwgaLjabdghaXbqabaGccqGGVaWlcqWGdbWqcqWGSbaBaeaacqWGdbWqdaWgaaWcbaGaeGimaadabeaakiabg2da9iabdseaejabd+gaVjabdohaZjabdwgaLjabc+caViabdAfawnaaBaaaleaacqWGLbqzcqWGXbqCaeqaaaaaaaGccaWLjaGaaCzcamaabmaabaGaeGOnaydacaGLOaGaayzkaaaaaa@598A@

where V_eq _is the equilibrium volume of distribution (determined from the tissue/blood partition and volume of each tissue) and Cl is the liver clearance. A quantitative measure of the influence of the liver clearance on the PBPK model kinetics is shown in fig. 14. This figures compares the kinetics for the simple one compartment model (eq. (6)) with the N = 1 and N = 3 PBPK models for cannabinol as a function of the liver clearance. (Since cannabinol has a K_oil-wat _of 257,000, its tissue/blood partition should be identical to that of the persistent solutes, eq. (5)). For a clearance of 1 l/min (the optimal model fit to the experimental cannabinol data (fig. 12)), the blood concentration has a strong dependence on the adipose flow model. For example, at 20 days, the residual blood cannabinol concentration for the N = 3 adipose model is about 10 times higher than for the N = 1 model and 100 times higher than the one compartment model, and this difference grows exponentially with time (fig. 14, upper left). However, if the liver clearance is reduced by a factor of 100 to 0.01 l/min, the liver clearance becomes rate limiting and, after about 10 days, the kinetics for the N = 3 and N = 1 PBPK model and the one compartment model (eq. (6) become nearly identical (fig. 14, lower right). The one compartment time constant (T) for a clearance of 0.01 l/min is 86 days (V_eq _= 1237 l), much longer than the time constant of the slowest adipose tissue compartment (6.26 days) of the N = 3 model. Since the half time of most persistent lipophilic compounds is a year or longer [47-50], the simple one compartment model (eq. (6)) provides a good approximation to their long time pharmacokinetics. The equivalence of the PBPK and one compartment models for a clearance of 0.01 l/min is only valid at long times. As shown in the inset in fig. 14, the one compartment model differs significantly from the N = 3 PBPK model for the first 10 days. If one is interested in modeling the early time kinetics after, e.g., a sudden exposure to dioxin, it is necessary to use the complete PBPK model with heterogeneous adipose blood flow.

### Flow limited versus diffusion limited adipose models

It is assumed in the PKQuest PBPK model that the exchange between adipose tissue and blood is flow (perfusion) rate limited. That is, the blood at the venous end of the capillary equilibrates with the well mixed tissue concentration so that the only parameter characterizing the exchange is the adipose perfusion rate. A number of groups have used a more complicated model in which the rate of adipose-blood exchange is characterized by both the perfusion rate and a diffusion limited permeability coefficient [16-19]. Flow limited models have the major advantage that one can use the perfusion rates determined from one solute to predict the pharmacokinetics of any other solute (if the adipose/blood partition coefficient is known). This cannot be done with the diffusion limited model since the permeability coefficient will vary from solute to solute. The ratio of the rates of blood/tissue exchange by diffusion and perfusion is qualitatively described by:

PerfusionDiffusion α FcBDWcW α Kbld−watF/DW     (7)
 MathType@MTEF@5@5@+=feaafiart1ev1aaatCvAUfKttLearuWrP9MDH5MBPbIqV92AaeXatLxBI9gBaebbnrfifHhDYfgasaacH8akY=wiFfYdH8Gipec8Eeeu0xXdbba9frFj0=OqFfea0dXdd9vqai=hGuQ8kuc9pgc9s8qqaq=dirpe0xb9q8qiLsFr0=vr0=vr0dc8meaabaqaciaacaGaaeqabaqabeGadaaakeaadaWcaaqaaiabdcfaqjabdwgaLjabdkhaYjabdAgaMjabdwha1jabdohaZjabdMgaPjabd+gaVjabd6gaUbqaaiabdseaejabdMgaPjabdAgaMjabdAgaMjabdwha1jabdohaZjabdMgaPjabd+gaVjabd6gaUbaacqqGGaaiiiGacqWFXoqycqqGGaaidaWcaaqaaiabdAeagjabdogaJnaaBaaaleaacqWGcbGqaeqaaaGcbaGaemiraq0aaSbaaSqaaiabdEfaxbqabaGccqWGJbWydaWgaaWcbaGaem4vaCfabeaaaaGccqqGGaaicqWFXoqycqqGGaaicqWGlbWsdaWgaaWcbaGaemOyaiMaemiBaWMaemizaqMaeyOeI0Iaem4DaCNaemyyaeMaemiDaqhabeaakiabdAeagjabc+caViabdseaenaaBaaaleaacqWGxbWvaeqaaOGaaCzcaiaaxMaadaqadaqaaiabiEda3aGaayjkaiaawMcaaaaa@6725@

where c_W _and c_B _are the water and blood concentration, F is blood flow and D_W _is the aqueous diffusion coefficient. (See the Additional file 2 for a detailed derivation of eq. (7)). The flow (perfusion) limited model assumes that perfusion is the slow, rate limiting process and the ratio in eq. (7) << 1. As the value of K_bld-wat _increases, the value of the ratio increases, increasing the relative importance of the diffusive component in limiting the rate of blood-tissue exchange.

One approach to determining if the diffusive component is limiting is to compare the pharmacokinetics of two solutes that have significantly different values of K_bld-wat _and, therefore, diffusion limitation (eq. (7)). If a flow limited model (with a fixed set of tissue flows) is able to accurately describe the kinetics of both solutes then this provides direct evidence that diffusion is not limiting either solute. As described in the Results, the 3 day cannabinol kinetics (fig. 12) can be accurately described by a flow limited model that used the identical set of adipose perfusion rates determined from modeling of the volatile anesthetic. Since the cannabinol K_bld-wat _is about 1000 times larger than that of the anesthetics (Tables 2 and 3), this provides direct qualitative support that these two solutes can be described by a flow limited PBPK model. Another argument that supports at least the qualitative validity of the flow limited model is the agreement between the average adipose blood flow determined using the PBPK flow limited model and direct methods using either Xe washout [39-43] or [^15^O]-labeled water [44]. A diffusion limitation, if it existed, should have different effects on these three methods and they should not agree.

There are two other factors that could produce departures from the simple flow limited model: 1) a direct arterial-venous shunt; and 2) intertissue diffusion from high blood flow tissues (eg. kidney, intestine, dermis) to surrounding adipose tissue [46,51]. Both of these effects also depend on the perfusion/diffusion ratio (eq. (7)) and the agreement between the anesthetic and cannabinol model results suggests that neither of them are important for these solutes. These model results provide qualitative support for the flow limited model for solutes with K_bld-wat _varying from 2 volatile anesthetics) to 2000 (cannabinol). Some PCBs have values of K_oil-wat _(and therefore, K_bld-wat_) that are as much as 100 times greater than that of cannabinol [52], with a corresponding Perfusion/Diffusion ratio 100 times greater (eq. (7)). These PCBs might have a significant diffusion limitation.

## Conclusion

The heterogeneous perfusion of human adipose tissue can only be resolved by following the blood concentrations for long times (2 days or more). The volatile anesthetic pharmacokinetic studies of Eger and colleagues [4,5] represents the most accurate and complete long term data set in the literature. The PBPK modeling of this data clearly indicates there are at least 2 different adipose compartments with perfusion rates varying by a factor of 5 or more. Although this heterogeneity significantly influences the long time pharmacokinetics of drugs such as cannabinol or tetrahydrocannibinol that have a relatively high liver clearance, it has an insignificant long time effect on persistent compounds such as the dioxins or PCBs that have very slow rates of clearance. Comparison of the model analysis of the volatile anesthetics versus cannabinol provides direct qualitative support for the validity of a flow limited adipose tissue PBPK model.

## Competing interests

The author(s) declare that they have no competing interests.

## Pre-publication history

The pre-publication history for this paper can be accessed here:



## Supplementary Material

Additional file 1 Average tabulated data for sevoflurane [5] and desflurane [4] studies. Click here for file

Additional file 2 Section I. Quantization of diffusion limitation. Section II. Factors determining the capillary permeability and a qualitative test of diffusion limitation. Section III. Prediction of oil/water partition coefficient using octanol/water coefficient. Click here for file
