# Supplementary material for: Heterogeneity of human adipose blood flow
Source: BMC Clin Pharmacol. 2007 Jan 20;7:1. doi: 10.1186/1472-6904-7-1 (PMC1797001; doi:10.1186/1472-6904-7-1)
Supplement: Additional file 2 — Section I. Quantization of diffusion limitation. Section II. Factors determining the capillary permeability and a qualitative test of diffusion limitation. Section III. Prediction of oil/water partition coefficient using octanol/water coefficient. [file 1472-6904-7-1-S2.doc]

**Diffusion limitation and oil-water partition.**

(Additional file 2 for “Heterogeneity of human adipose blood flow” by David G. Levitt)

**I. Quantization of diffusion limitation.**

**Notation:**

v_i_ = weight (Kg) of organ i

C_i_ = total concentration (mole/cm^3^) in organ i.

c_i_ = free concentration in water (moles/cm^3^)

F = organ blood flow (Kg/sec)
P = permeability (cm/sec)

a = capillary radius (cm)

N = capillary density (#/cm^3^)

S = capillary surface area surface area per cm^3^ tissue (1/cm) (=2πaN)

Clr = fraction of solute that equilibrates across capillary in one pass

K_bld-wat_ = Blood/water partition coefficient

K_oil-wat =_ Oil/water partition coefficient.

D_w_ and D_L_ = Diffusion coefficient in water and lipid (cm^2^/sec).

L = Thickness of unstirred water layer separating blood and fat tissue.

f_L_ = Fraction of blood or tissue that is lipid.

This first section assumes that the blood-tissue exchange can be described by an “equivalent” capillary permeability that characterizes the exchange between the blood and a well-stirred tissue concentration. Section II describes how this permeability can be related to the solute diffusion coefficient.

**Tissue**

**c_T_ C_T_**

**F**

**C_A_ c_A_**

**c_v_ C_v_**

**c_B_(x)**

**c(x)**

**x**

The above figure describes the exchange of solute between the blood and tissue compartment across the capillary wall. It is assumed that the tissue consists of N capillaries/kg, all with exactly the same geometry, blood flow, permeability, etc. It will also be assumed that the relation between the capillary and tissue concentration is in a pseudo steady state. The steady state differential equation for the concentration in the capillary as a function of position is:

$-(F/v)\frac{dC_{B}}{dx}=2\pi aPN[c_{B}(x)-c_{T}]$

where C and c are the total and free water concentration, subscript B and T refers to the blood and tissue compartment, P is the capillary permeability, N is the number of capillaries per kg tissue, and a is the capillary radius. The total concentration C_B_ can be related to the free water concentration (c_B_) using the blood/water partition coefficient (C_B_ = K_bld-wat_ c_B_). Integrating eq. 1 over the length of the capillary and solving for the venous concentration leaving the capillary:

$$c_{v}=c_{T}+[c_{A}-c_{T}]\exp(-(PS/K_{bld-wat})(v/F))$$

where PS is the permeability-surface area product per tissue weight (liters/min/Kg). Equation 2 can be related to fractional clearance or equilibration that occurs in one pass through the capillary:

$$\begin{matrix} Clr=(c_{A}-c_{V})/(c_{A}-c_{T})=1-\exp(-\kappa) \\ \kappa= (PS/K_{bld-wat})(v/F) \end{matrix}$$

For κ>>1, Clr approaches 1 and the solute is flow limited. The clearance depends on both the intrinsic permeability (P) and the blood-water partition coefficient. Solutes that have a high intrinsic permeability may be permeability (or diffusion) limited if a large fraction of the blood solute is bound (i.e. large K_bld-wat_).

**II. Factors determining the capillary permeability and a qualitative test of diffusion limitation.**


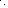


**C↔c_W_**

**D_W_**

**c_L_ ↔ c_W_**

**D_L_**

**C_art_**

**C_vein_**

**F**

**Fat Tissue**

**D_W_**

**L**

The above figure shows a schematic diagram of the processes involved in the exchange of solute between the blood and adipose (“fat”) tissue. There are two barriers in series limiting the diffusive exchange between the blood and adipose tissue: 1) an inner aqueous region of equivalent thickness L (blood, capillary wall and extravascular space), and 2) an outer lipid region. Because of the radial geometry of the capillary-tissue system, most of the resistance to diffusion is in the first 5 to 10 microns surrounding the capillary. The relative rates of diffusion through the aqueous and lipid regions are:

$$\begin{matrix} Aqueous Diffusion \alpha D_{w}c_{w} \\ Lipid Diffusion \alpha D_{L}c_{L} = K_{oil-wat}D_{L}c_{w} \end{matrix}$$

The ratio of the lipid/aqueous diffusion rate is:

$$Lipid/Aqueous Diffusion = K_{oil-wat}D_{L}/D_{w}$$

The following table lists the values of the partition and diffusion coefficients for the 5 solutes modeled in the paper. The partition values for the volatile anesthetics are experimental. For the cannabinoids, the oil-water partition is derived from the experimental value of their octanol/water partition (see Section III) and the blood/water and oil/blood partitions are derived using eqs. 3 and 4 of the main text. The aqueous diffusion coefficients are based on using the Stokes-Einstein relation with glucose as a standard (D_x_ = D_glucose_[mol. wt glucose/ mol. wt. X]^1/3^ ). The lipid diffusion coefficients are based on the following estimates. Since the viscosity of olive oil is about 85 times that of water, the Stokes-Einstein relation predicts the diffusion coefficient should be 85 times smaller in oil. However, this relation is valid only if the solute is much larger than the solvent, which is not true for diffusion in olive oil. For small solutes, such as O_2_ or N_2_, the diffusion coefficient in olive oil is only about 3 times smaller than in water [1]. Rogacheva et. al. [2] measured diffusion coefficients of 2-nonanone and benzaldehyde in oil that were about 10 times less than the water value. The value of 20 used in the table for D_W_/D_L_ is just a rough estimate. The next to last column in the table lists the lipid/water diffusivity ratio (eq. ) which determines the relative importance of the aqueous versus lipid diffusion. For the highly lipid soluble solutes such as cannabinol or cannabidiol, the lipid diffusivity is 1000 to 10000 times larger than the aqueous diffusivity. This implies that it is the diffusion through the aqueous region (thickness L, above figure) that is the rate limiting step in diffusive exchange between the blood and adipose tissue.

| Solute | K_oil-wat_ | K_bld-wat_ | K_oil-bld_ | D_W_  (cm^2^/sec) | D_W_/D_L_ | K_oil-wat_D_L_/D_W_ | D_W_/K_bld-wat_ |
| --- | --- | --- | --- | --- | --- | --- | --- |
| Isoflurane | 162 | 2.18 | 74 | 0.66x10^-5^ | ≈20 | 8.1 | 0.3x10^-5^ |
| Sevoflurane | 138 | 1.82 | 76 | 0.65x10^-5^ | ≈20 | 6.9 | 0.35x10^-5^ |
| Desflurane | 79.5 | 2.31 | 34.4 | 0.68x10^-5^ | ≈20 | 3.97 | 0.3x10^-5^ |
| cannabinol | 257,000 | 1927 | 133 | 0.56x10^-5^ | ≈20 | 12850 | 0.29x10^-8^ |
| cannabidiol | 24,000 | 180 | 132 | 0.56x10^-5^ | ≈20 | 1200 | 0.3x10^-7^ |

As described in section I, the degree of diffusion limitation for a given flow (F) and capillary permeability (PS) is characterized by the parameter PS/K_bld-wat_ (eq. ). It is not possible to theoretically estimate this parameter because of large uncertainties in the factors that determine P (e.g., value of aqueous thickness L, tissue geometry, mixing, diffusion coefficient, etc.). However, one can qualitatively test for a diffusion limitation by comparing the pharmacokinetics of solutes with different values of PS/K_bld-wat_. For example, if, as discussed above, diffusion trough the aqueous region (thickness L) is the rate limiting diffusion barrier, then P is proportional to D_W_, and the diffusion limitation is proportional to D_W_/K_bld-wat_. This parameter is listed in the last column of the above table. Its value for cannabinol is about 1000 times smaller then for the volatile anesthetics. If the blood/adipose exchange was not perfusion limited (i.e. some diffusion limitation), then one would predict that the pharmacokinetics of cannabinol and the volatile anesthetics could not be fit using the same adipose blood flow and flow limited model.

**III. Prediction of oil/water partition coefficient using octanol/water coefficient.**

There are no available experimental values of the oil/water (e.g. olive oil/water) partition coefficient for cannabinol or cannabidiol. However, it will be shown here that for solutes with these relatively simple structures one can predict (within a factor of about 2) the oil/water partition from the known value of the octanol/water partition. Figure 1 (below) shows a plot of (log octanol/water - log oil/water partition) versus the log octanol/water partition for a series of non-polar solutes. Figure 2 shows a similar plot for solutes with 1 or 2 aliphatic or aromatic hydroxyl groups and no other polar groups. The oil/water data in these figures is based on values from the literature [3-14]. The octanol/water data are the recommended values from the web site of James Sangster (<http://logkow.cisti.nrc.ca/logkow/intro.html>).

It can be seen in fig. 1 that for the non-polar solutes, the oil-water and octanol-water partition are nearly identical (octanol-water partition about 25% less then oil-water) over a very wide range of structures. As shown in fig. 2, the addition of 1 aliphatic hydroxyl makes the oil-water partition about 1.03 log units (factor of 10.5) smaller than the octanol-water partition and one aromatic hydroxyl makes the oil-water about 0.82 log units (6.6) smaller. The larger partition of the hydroxyl compounds in octanol versus olive oil results from the hydrogen bond forming potential of octanol. The data in fig. 2 also show that the effect of adding additional hydroxyls is roughly additive.

Using the experimental values for the log octanol/water partition coefficients for cannabinol (log Koct-wat = 6.23) and cannabidiol (log Koct-wat = 5.79) [15], the predicted log Koil-wat values for cannabinol is 5.41 (Koil-wat = 257,000) (one aromatic hydroxyl) and for cannabindiol is 4.38 (Koil-wat = 24,000) (two aromatic hydroxyls). These are the values that are used in Table 4 of the main paper.

**References**

1. Davidson D, Eggleton P, Foggie P: **The diffusion of atmospheric gases through fats and oils**. *Q J Exp Physiol Cogn Med Sci* 1952, **37**(2):91-105.

2. Rogacheva S, Espinosa-Diaz MA, Voilley A: **Transfer of aroma compounds in water-lipid systems: binding tendency of beta-lactoglobulin**. *J Agric Food Chem* 1999, **47**(1):259-263.

3. Chiou CT: **Partition coefficients of organic compounds in lipid-water systems and correlations with fish bioconcentration factors**. *Environ Sci Technol* 1985, **19**:57-62.

4. Jabusch TW, Swackhamer DL: **Partitioning of polychlorinated biphenyls in octanol/water, triolein/water, and membrane/water systems**. *Chemosphere* 2005, **60**(9):1270-1278.

5. Gargas ML, Burgess RJ, Voisard DE, Cason GH, Andersen ME: **Partition coefficients of low-molecular-weight volatile chemicals in various liquids and tissues**. *Toxicol Appl Pharmacol* 1989, **98**(1):87-99.

6. Meulenberg CJ, Wijnker AG, Vijverberg HP: **Relationship between olive oil:air, saline:air, and rat brain:air partition coefficients of organic solvents in vitro**. *J Toxicol Environ Health A* 2003, **66**(20):1985-1998.

7. Kaneko T, Wang PY, Sato A: **Partition coefficients of some acetate esters and alcohols in water, blood, olive oil, and rat tissues**. *Occup Environ Med* 1994, **51**(1):68-72.

8. Oldendorf WH: **Lipid solubility and drug penetration of the blood brain barrier**. *Proc Soc Exp Biol Med* 1974, **147**(3):813-815.

9. Fiserova-Bergerova V, Tichy M, Di Carlo FJ: **Effects of biosolubility on pulmonary uptake and disposition of gases and vapors of lipophilic chemicals**. *Drug Metab Rev* 1984, **15**(5-6):1033-1070.

10. Sato A, Nakajima T: **Partition coefficients of some aromatic hydrocarbons and ketones in water, blood and oil**. *Br J Ind Med* 1979, **36**(3):231-234.

11. Sato A, Nakajima T: **A structure-activity relationship of some chlorinated hydrocarbons**. *Arch Environ Health* 1979, **34**(2):69-75.

12. Eger EI, 2nd, Ionescu P, Laster MJ, Gong D, Hudlicky T, Kendig JJ, Harris RA, Trudell JR, Pohorille A: **Minimum alveolar anesthetic concentration of fluorinated alkanols in rats: relevance to theories of narcosis**. *Anesth Analg* 1999, **88**(4):867-876.

13. Zhang Y, Trudell JR, Mascia MP, Laster MJ, Gong DH, Harris RA, Eger EI, 2nd: **The anesthetic potencies of alkanethiols for rats: relevance to theories of narcosis**. *Anesth Analg* 2000, **91**(5):1294-1299.

14. Leo A, Hansch C, Elkins D: **Partition coefficients and their uses**. *Chemical Reviews* 1971, **71**(6):525-616.

15. Thomas BF, Compton DR, Martin BR: **Characterization of the lipophilicity of natural and synthetic analogs of delta 9-tetrahydrocannabinol and its relationship to pharmacological potency**. *J Pharmacol Exp Ther* 1990, **255**(2):624-630.


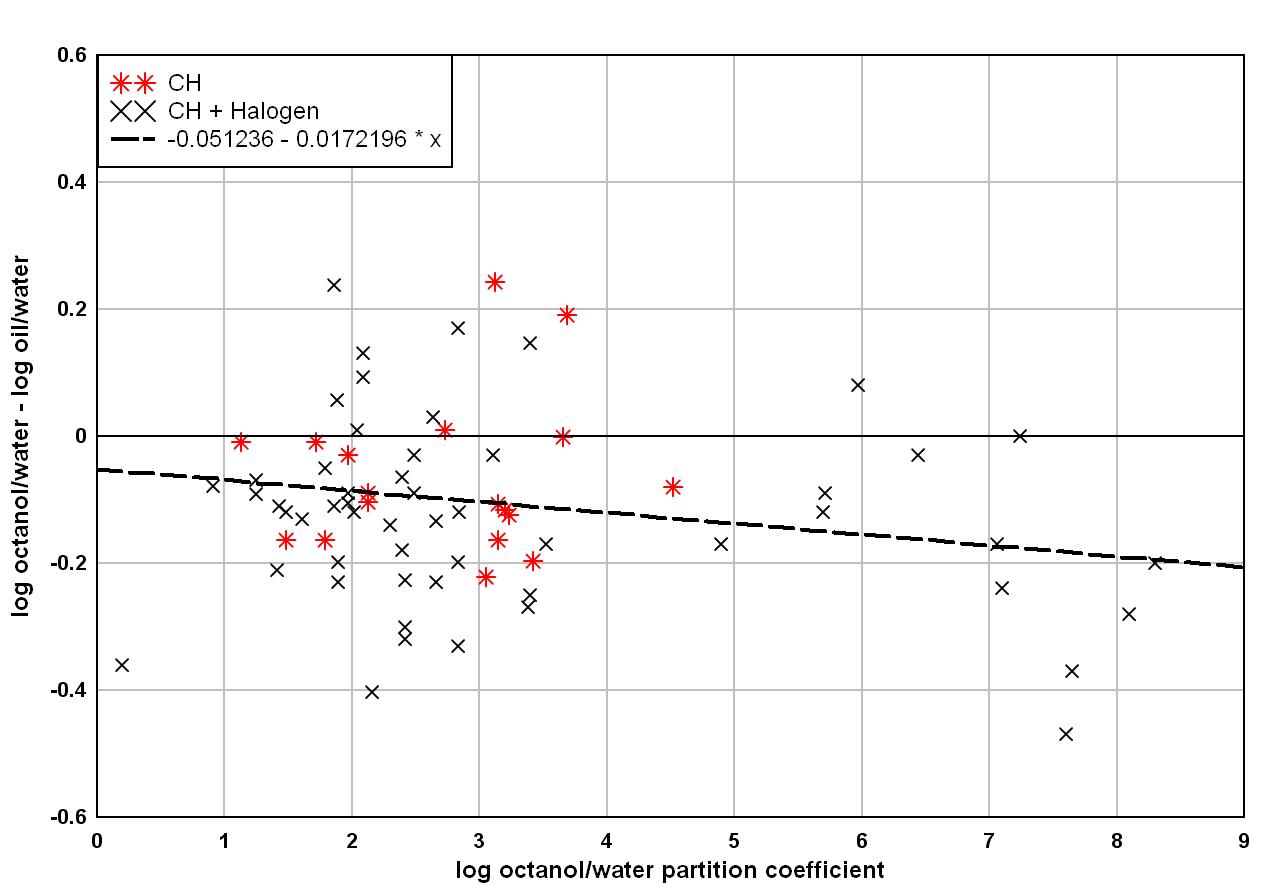


Figure 1. Plot of (log octanol/water – log oil/water) versus log octanol/water for non-polar solutes. Pure hydrocarbon molecules (*) and halogenated hydrocarbons (x) are indicated. The dashed line is the least square fit to all the data.


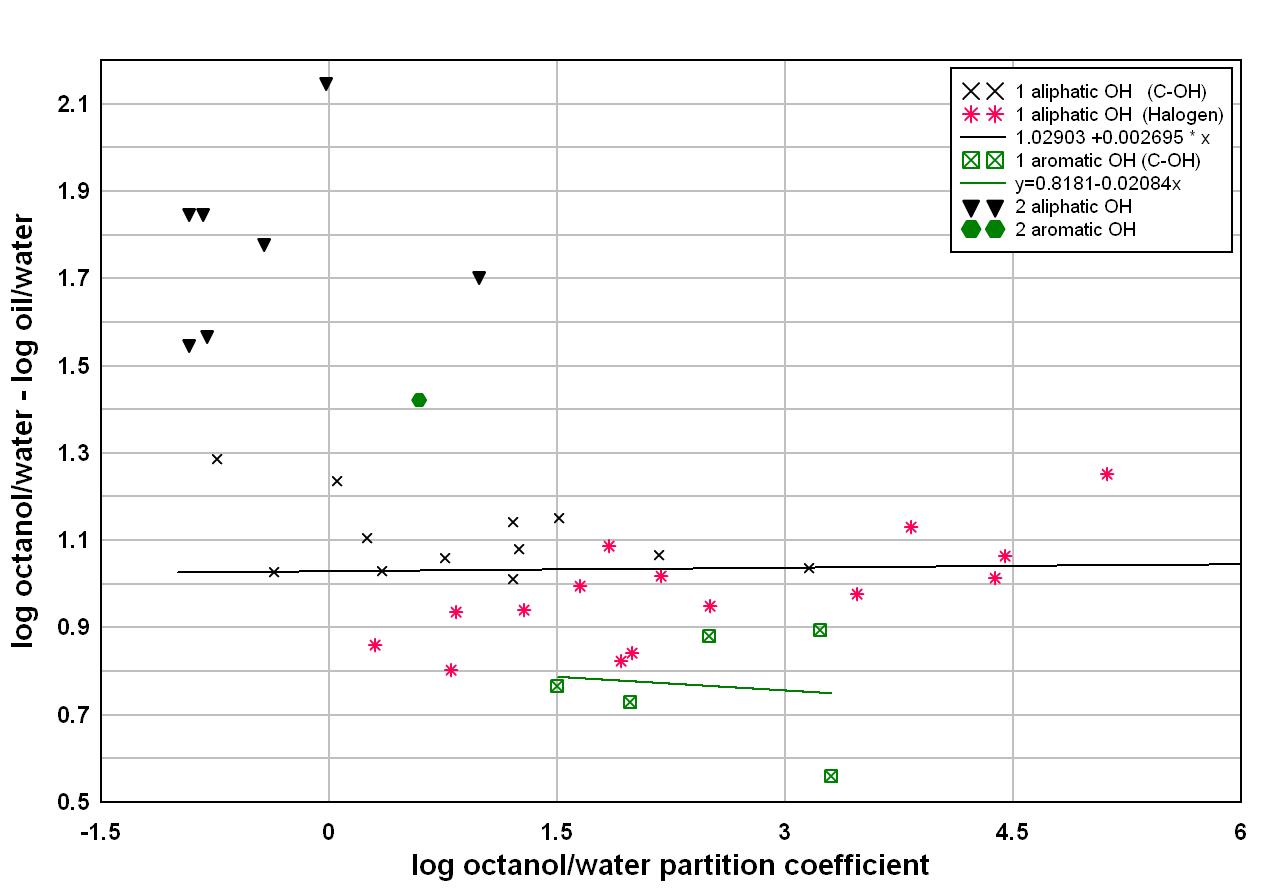


Figure 2. Plot of (log octanol/water – log oil/water) versus log octanol/water for solutes with 1 or 2 hydroxyl groups.
